# Prevalence of Microalbuminuria in Type 2 Diabetes Mellitus

**DOI:** 10.7759/cureus.12318

**Published:** 2020-12-27

**Authors:** Muhammad Ahsan Sana, Manahil Chaudhry, Ayesha Malik, Noreena Iqbal, Ayesha Zakiuddin, Mohammad Abdullah

**Affiliations:** 1 Medicine, CMH Lahore Medical College and Institute of Dentistry, Lahore, PAK; 2 Medicine, Hameed Latif Hospital, Lahore, PAK; 3 Medicine, Hameed Latif Hospital, Lahore , PAK; 4 Internal Medicine, Milton Keynes University Trust Hospital, Milton Keynes, GBR

**Keywords:** diabetes mellitus type 2, microalbuminuria, diabetic nephropathy (dn)

## Abstract

Objective: The presence of albumin in the urine is a marker of glomerular involvement in type 2 diabetes mellitus (T2DM), depicting diabetic nephropathy. Strict glycemic control can prevent and delay the occurrence of microalbuminuria and other diabetic complications. Therefore, we conducted a study to report the prevalence of microalbuminuria in type 2 diabetics along with its association with diabetic control.

Methods: A total of 133 patients with T2DM were consecutively included and their co-morbidities, body mass index, mode of treatment of diabetes (oral hypoglycemic drugs and/or insulin), duration since diagnosis of T2DM, and hemoglobin A1c (HbA1c) levels were recorded. A morning, mid-stream urine sample was collected and a urine spot for albumin:creatinine ratio (UACR) was assessed. Descriptive and analytic statistics were drawn with different variables and UACR values.

Results: The mean age of the participants was 54.5 ± 10.3 years which included 60.9% males and 39.1% females. The overall incidence of diabetic nephropathy was 30.1%, with 25.6% having microalbuminuria and 4.5% having macroalbuminuria. Pearson correlation test was used to compare UACR and duration of diabetes (p=0.034) and HbA1c (p=0.001).

Conclusion: UACR was higher in patients with uncontrolled T2DM (in terms of higher HbA1c value) and with a longer duration since diagnosis. We recommend that UACR should be inculcated in routine practice, annually, for all patients with T2DM for gauging the development of underlying renal involvement and prompt management.

## Introduction

The prevalence of type 2 diabetes mellitus (T2DM) is increasing worldwide and so are the disease-associated complications [1]. Long-term complications of diabetes cause significant morbidity and mortality. Complications of diabetes can be macrovascular and/or microvascular. Macrovascular complications include myocardial infarction, transient ischemic attack, stroke, and limb ischemia and microvascular complications include retinopathy, nephropathy, peripheral neuropathy, and autonomic neuropathy. Adequate glycemic control is important to delay or prevent these complications.

Diabetic nephropathy is one of the most common causes of chronic kidney disease (CKD) leading to end-stage renal disease (ESRD) and its prevalence is increasing because of the increasing burden of T2DM [2]. For early detection of diabetic nephropathy, the American Diabetic Association (ADA) recommends screening for microalbuminuria once a year for diabetic patients [3]. Previously, a 24-hour urine collection was used for measurement of urinary albumin excretion. However, a spot morning sample for urinary albumin:creatinine ratio (UACR) is now used for screening of microalbuminuria. It is convenient and correlates well with 24-hour collection results in adults [3,4].

UACR has a direct relation to diabetic control and glycosylated hemoglobin (HbA1c) is a useful tool to assess diabetic control. HbA1c levels ≥ 6.5% are diagnostic for diabetes mellitus and levels < 7.0% are recommended in diabetic patients [5,6]. This study aims to find the prevalence of microalbuminuria and its correlation with diabetic control, represented by the HbA1c, in patients with T2DM.

## Materials and methods

This cross-sectional study was conducted from January 2020 to June 2020 at a tertiary care hospital after acquiring permission from the hospital’s ethical review board. Informed consent was sought from all patients who met the inclusion criteria and were consecutively included.

A total of 133 diabetic patients, 18 years or older, visiting the outdoor patient department (OPD) were included. Previously diagnosed cases of T2DM on an oral hypoglycemic drug(s) and/or insulin treatment were included. Amongst the clinic attendees those having an active infection, fever, congestive heart failure, recent or current pregnancy, history of vaginal discharge, known cases of chronic kidney disease (CKD), those diagnosed as nephrotic syndrome, and patients reporting after vigorous exercise were excluded from the study. Patients with type 1 diabetes mellitus were also excluded.

Patients were interviewed and demographic and clinical data such as age, sex, duration since diagnosis of diabetes, history of ischemic heart disease and hypertension, smoking, and medication history were recorded. Weight, height, and blood pressure were measured during the clinic visit. Body mass index (BMI) was calculated by dividing weight in kilograms by the square of the height in meters. The patients were instructed to give a clean catch, mid-stream urine sample from their first morning void on the day following the visit, and the UACR ratio was requested. Microalbuminuria was defined as having a UACR ≥ 3.5 mg/mmol (but < 30 mg/mmol) in females and ≥ 2.5 mg/mmol (but < 30 mg/mmol) in males, while macroalbuminuria was defined as having UACR more than 30 mg/mmol. These were the primary end-points of identifying diabetic nephropathy. Simultaneously, an HbA1c level was also acquired. A 2 ml peripheral venous sample was collected in a test tube with ethylene diamine tetraacetic acid (EDTA) and sent for evaluation at the hospital’s laboratory by the immunoturbidity method. This was optically measured and was a quantitative analysis. Levels < 7.0% were considered as well-controlled diabetes mellitus while those above 7.0% were considered as poorly controlled diabetes mellitus. Data were analyzed using Statistical Package for Social Sciences (SPSS) Statistics version 25 (IBM Corp., Armonk, NY, USA).

## Results

Out of the total 133 study participants, 60.9% (n=81) were male and 39.1% (n=52) were female. Mean age was 54.5 ± 10.3 years and mean duration of diabetes was 6.29 ± 6.27 years, mean HbA1c was 8.99 ± 2.03%, and mean BMI was 26.75 ± 3.73 kg/m2. Demographic and clinical features are described in Table 1. The overall incidence of diabetic nephropathy was 30.1% with 25.6% having microalbuminuria and 4.5% having macroalbuminuria (Table 1).

**Table 1:** Demographic and clinical features of the study population (n=133)

		Frequency	Percentage
Hypertension		56	42.1%
Ischemic heart disease		12	9.0%
Smoker		38	28.6%
BMI	Underweight	0	0.0%
	Normal	50	37.6%
	Overweight	57	42.9%
	Obese	26	19.5%
Albuminuria	Yes	40	30.1%
	No	93	69.9%
Insulin (alone)		8	6.0%
Oral hypoglycemic drug		98	73.7%
Insulin + oral hypoglycemic drug		27	20.3%

A Pearson product-moment correlation coefficient was computed to assess the relationship between UACR and HbA1c and UACR and duration of diabetes. There was a positive correlation between HbA1c and UACR (r=0.292, p=0.001). A positive correlation was also established between duration of diabetes and UACR (r=0.183, p=0.035) hence proving that UACR was higher in patients with uncontrolled diabetes (in terms of higher HbA1c value) and longer duration of diabetes.

## Discussion

Globally, the prevalence of T2DM is on the rise. In a report estimating the worldwide burden of diabetes, King et al. reported an expected rise of 122% in the incidence of diabetes between 1998 and 2025 [7]. Local statistics show the mortality of around 120,000 patients, annually, owing to diabetes-related complications [8]. As per the National Health and Nutrition Examination Survey (NHNES), kidney disease was the most prevalent complication (27.8%) between 1998 and 2004 in diabetic patients [9].

Microalbuminuria is an early predictor and a sensitive assay to detect urinary albumin excretion which can precede the development of overt nephropathy in T2DM. Prompt detection and treatment can reduce the risk and possibly delay the development of ESRD. The American Diabetes Association recommends annual screening for microalbuminuria in patients of T2DM [4]. However, due to poor healthcare infrastructure and lack of education, people are not routinely screened in developing or underdeveloped countries and end up presenting late in the disease course. 

The prevalence of diabetic nephropathy in T2DM patients in our study was 30.1%, with 25.6% having microalbuminuria and 4.5% having macroalbuminuria. Some local studies stated the following percentages: Ahmad et al. reported 31.56% diabetic patients with microalbuminuria [10] and Muhammad et al. reported an overall microalbuminuria prevalence of 32.9% [11]. Additionally, a study by Anwarulla et al. spotted microalbuminuria in 33% of type 2 diabetics [8]. Within the subcontinent, a Bangladesh-based study reported a prevalence of microalbuminuria among diabetic participants of 29.72% [12]. Kanakamani et al. reported 25.5% of type 2 diabetics in North India with microalbuminuria [13] and Thakur et al. reported that 20% of Nepalese diabetic patients had microalbuminuria [14].

Some of the factors that influence kidney disease development include genetics, blood sugar control, and blood pressure. The impact of strict diabetic control on prognosis is most pronounced in patient’s microalbuminuria [9]. We found a significant correlation between higher HbA1c level and presence of microalbuminuria. This was synonymous with the results reported by Showail et al., Amini et al., Al-Shaikh et al., and Patel et al. [15-18].

We identified some limitations of our study. Firstly, it was a small sample size. In addition, a detailed drug history, as well as a history of dietary protein intake before sample collection, was not taken into account. These can be confounding factors that may alter the UACR value.

## Conclusions

A leading precedent of ESRD is diabetic nephropathy. Both early detection and progress of the disease can be determined by sensitive markers like the UACR. In addition, our study shows that poorly controlled diabetes is associated with higher UACR. Involvement of the UACR in routine practice should be considered for all diabetic patients.
